# Does Exposure to Summer Season at Different Stages of Intrauterine Development and Maternal Parity Affect Health and First-Lactation Milk Production of Female Offspring of Holstein Cows? †

**DOI:** 10.3390/ani14203040

**Published:** 2024-10-21

**Authors:** Hamed Beiranvand, Abolfazl Mahnani, Ali Kahyani, Frank R. Dunshea, Farhad Ahmadi

**Affiliations:** 1 R&D Department of Chaltasian & Talise Asil Jahan Agro-Animal Husbandry, Varamin 33751-13111, Iran; 2 Department of Animal Sciences, College of Agriculture, Isfahan University of Technology, Isfahan 84156-83111, Iran; 3 Department of Animal Science, School of Agriculture, Shiraz University, Shiraz 71441-65186, Iran; 4 School of Agriculture, Food and Ecosystem Sciences, Faculty of Science, The University of Melbourne, Parkville, VIC 3010, Australia; fdunshea@unimelb.edu.au; 5 Faculty of Biological Sciences, The University of Leeds, Leeds LS2 9JT, UK

**Keywords:** developmental programming, gestation, heifer, herd life, hyperthermia

## Abstract

A suboptimal intrauterine environment during gestation may result in the programming of long-lasting structural and physiological alterations in the developing fetus, leading to health and production complications in adulthood. This observational study aimed to identify the impact of exposure to the summer season at different trimesters of gestation, dam parity, and their interaction on the postpartum disease incidence, first-lactation milk production, and herd lifespan of the offspring (F_1_ generation). Using a dataset collected from two commercial herds, the female offspring were categorized into three groups based on the trimester their dams experienced summer season during pregnancy: (1) first trimester (n = 2345), (2) second trimester (n = 3513), and (3) final trimester (n = 4988). The estimated 305-day milk production was lower in daughters (as a first-lactation cow) born to dams exposed to summer season during the first vs. third trimester. Summer season exposure during the first vs. third trimester resulted in the offspring that were less likely to remain in the herd (hazard ratio = 0.90; 95% CI = 0.84–0.95). Daughters of parous vs. nulliparous dams were more likely to experience dystocia and metritis but less likely to experience retained placenta. The risk of culling was higher in daughters of parous vs. nulliparous dams. Our preliminary findings suggest that the first trimester is a critical determinant of the female progeny’s future productive performance and survivability. Dam parity was also identified as an influential factor affecting offspring health, as dystocia and metritis were more prevalent, and the culling risk was greater in daughters born to parous dams.

## Simple Summary

Heat stress during pregnancy can negatively affect the intrauterine environment, compromising fetal development and resulting in long-term physiological and structural changes that impact the postnatal outcomes of the offspring. The aim of this retrospective longitudinal study was to identify how exposure to summer season at different trimesters of intrauterine development and dam parity affect postpartum disease incidence, milk production, and herd lifespan of the offspring (F_1_ generation) of Holstein cows. Our preliminary findings confirmed less milk production capacity and greater culling risk in offspring experiencing the summer season during the first trimester of their intrauterine development than those during later stages. Daughters born to nulliparous dams had a lower risk of dystocia and metritis, a higher risk of retained placenta, and were more likely to remain in the herd than those born to parous dams. Minimizing summer heat exposure during the early intrauterine development of offspring through implementing heat-abatement strategies may help alleviate the risk of future production losses.

## 1. Introduction

Intrauterine condition is important for proper fetal development, enabling the offspring to express their full genetic potential in adulthood [1,2]. In response to maternal or environmental stressors, the developing embryo and fetus exhibit a high degree of plasticity in modifying their development, which may induce permanent structural, physiological, and metabolic changes [3,4]. Neonates born alive with compromised development may face lifelong complications in their health, growth, production, and reproductive performance in adulthood [5,6,7]. Heat stress is a major stressor for livestock [8]. The dairy industry suffers from escalating economic losses due to heat stress, which is predicted to worsen according to the climatic projections [9]. Exposure to increased environmental temperatures may trigger a cascade of physiological and behavioral changes that directly affect the metabolism, production, and reproductive performance of lactating cows [10]. Indirectly, maternal heat stress may result in detrimental, long-lasting effects on the postnatal health and performance of the offspring in adulthood [11].

Until birth, the fetal temperature is intricately connected to maternal regulation, implying that any fluctuations occurring in the dam’s core temperature may potentially be experienced by the developing fetus [12]. Elevated body temperature during pregnancy may disturb the intrauterine environment, as blood flow to the uterus is decreased and diverted to peripheral tissues [13]. Compromised transport of nutrients and oxygen due to heat stress may induce placental inefficiency, which is associated with impaired fetal development, leading to long-lasting structural and functional changes in offspring, associated with both short- and long-term epigenetic modifications [12,14]. These prenatal developmental alterations may negatively affect the offspring’s growth, development, and health in adulthood [3]. Laporta et al. [14] conducted a comprehensive 10-year analysis of maternal heat stress during the late gestation (the last 46 d) with multiparous Holstein cows in Florida, USA. Their experimental design involved comparing cows exposed to heat stress (provided only with shade) to those receiving active cooling (shade from a freestall barn, fans, and water soakers), and their findings suggested that the heat stress exposure had negative carryover effects on survivability and milk production of at least two subsequent generations. The authors theorized that prenatal heat stress exposure could alter the trajectory of development, causing less metabolically efficient phenotypes to emerge, ultimately compromising lifetime performance [14]. Female offspring born to Holstein dams experiencing the summer season at early gestation (conceived in the summer) had inferior subsequent survivability and produced less milk (258, 76, and 57 kg over a 305-day lactation period in parities 1, 2, and ≥3, respectively) than their counterparts conceived in the winter [3]. Comparatively less information is available in the literature about the postnatal outcomes of the offspring experiencing heat stress during their early intrauterine development. This stage is crucial, as organs begin to develop and establish typically within the first 90–180 days of intrauterine development [12]. This period is also particularly important in lactating pregnant dams, as it coincides with the lactation phase.

Maternal parity is an important factor in creating optimal intrauterine conditions, influencing early-life developmental programming and potentially determining the future health and production of the offspring [15]. For example, Bafandeh et al. [16] reported that maternal age (Holstein dairy cows classified from nulliparous dams to dams with seven or more parities) was inversely associated with the offspring’s lactation capacity. In contrast to nulliparous dams, the early- and mid-fetal development in parous, pregnant cows coincides with maternal lactation. This unique coincidence highlights the competition for feed nutrients between the developing fetus and mammary gland for milk synthesis [17], which becomes even more critical during heat stress when feed intake depressions usually occur [8].

Our hypothesis was that dam parity and exposure to summer season during different stages of intrauterine development would have different, long-lasting effects on the postnatal outcomes of the offspring in adulthood. Therefore, this retrospective longitudinal study was designed using a large dataset to track the potential impacts of (1) the timing of summer season exposure during early, mid, and late gestational stages and (2) maternal parity (nulliparous and parous) and their interaction on the health and production capacity of the female offspring (F_1_ generation) in their first lactation.

## 2. Materials and Methods

### 2.1. Description of Herds and Dataset Acquisition

This observational study did not involve the direct use of animals, and no animal experimental approval was required or obtained. The data used in this study consist of records on Holstein cows from two herds (A and B) located in the same region (Varamin, Tehran, Iran; 35°19′ N latitude and 51°41′ E longitude and an elevation of 927 m above sea level), approximately within a 10 km radius. Herd A belonged to the Chaltasian Agro-animal husbandry (with approximately 2300 lactating cows and an average 305-day milk production of 11,335 kg). Herd B belonged to the Taliseh Asil Jahan Agro-animal husbandry (about 2500 lactating cows and an average 305-day milk production of 11,107 kg). The characteristics and performance metrics of herds A and B are reported in Supplementary Table S1. Both herds were operated and managed under a similar management system, functioning as a hybrid entity and combining aspects of both a commercial and experimental organization. Records from dams (F_0_) and their daughters (F_1_) were retrieved from the farm management software program (Modiran Software (version 2.3), Isfahan, Iran). The data collected were dam information including their parity and date of conception, and the information of their offspring included the birth date and birth weight, age at first calving (AFC), postpartum diseases (dystocia, retained placenta, and metritis), estimated first-lactation 305-day milk yield, and culling date from the herd. AFC was computed as the calving date minus the birth date.

### 2.2. Study Design

**Figure 1:**
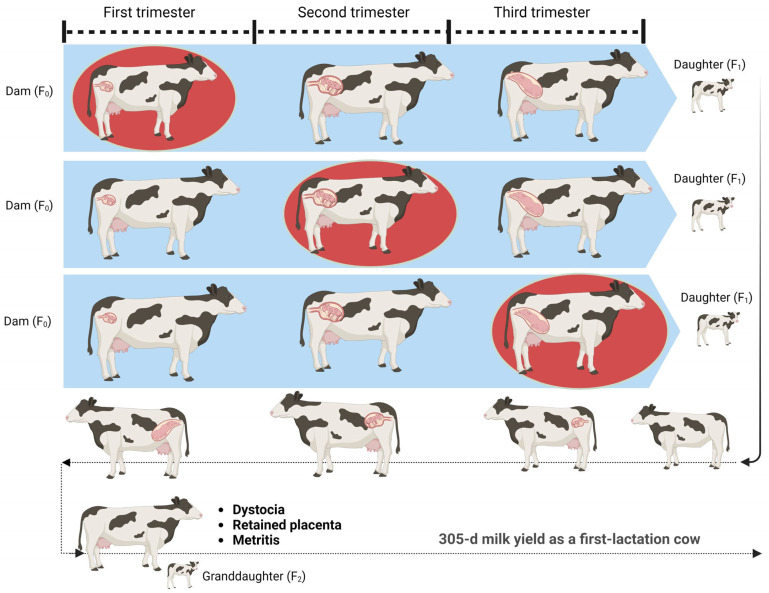
A graphical illustration of the experimental design. Pregnant Holstein cows were categorized into three groups according to their gestation phase exposure to the summer season (red oval-shaped): the first, second, and final trimester of gestation. The summer season was defined as days from June to September. Birth weight, first-calving age, the incidence of postpartum diseases, first lactation milk production, and the risk of culling were tracked in daughters of dams exposed to the summer season at different gestational phases. The figure was created using BioRender (https://biorender.com/, accessed on 5 September 2023).

The offspring (F_1_) were categorized into three groups according to the trimester when their dams were exposed to the summer season: (1) dams experiencing the first trimester of gestation in summer season (n = 2345), (2) dams experiencing the second trimester of their gestation in summer season (n = 3513), and (3) dams experiencing the final trimester of their gestation in summer season (n = 4988). A visual illustration of the whole experimental design is provided in Figure 1. The summer season was defined as days from June to September. All Freemartin offspring were excluded from the dataset. There were 10,848 data for female offspring in the initial dataset, along with 5066 first-lactation records (from 2013 to 2020).

### 2.3. Meteorological Data

**Figure 2:**
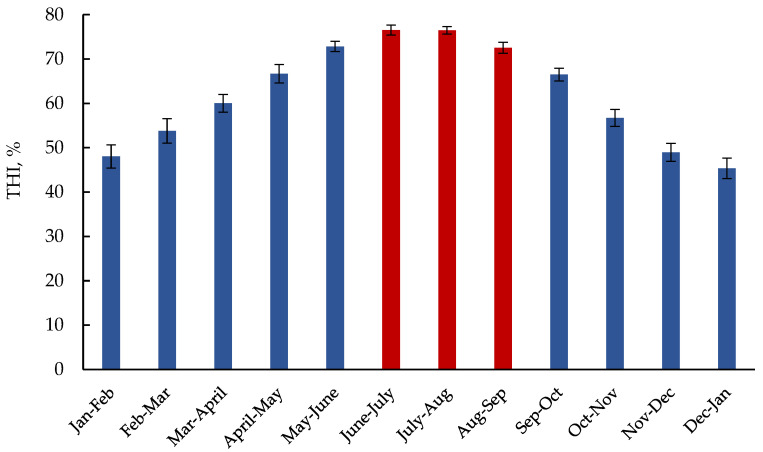
Monthly mean ± standard deviation of mean temperature–humidity index (THI) from 2013 to 2023.

Retrospective weather data were collected from the nearest local meteorological station (Iran Meteorological Organization), located approximately 9.4 km from herd A and 7 km from herd B. The temperature–humidity index (THI) was estimated as [0.8 × mean temperature + (mean relative humidity (%)/100) × (mean temperature − 14.4) + 46.4] [18]. The monthly mean THI from 2013 to 2023 is presented in Figure 2. A box-and-whisker plot of monthly average temperature and relative humidity from 2013 to 2023 (Supplementary Figure S1) was developed using Python code written in Jupyter Notebooks.

### 2.4. Management of Dams

Cows in both herds were kept in an intensive production system with free-stall barns. Diets were offered in the form of a total mixed ration. Both herds were fed a total mixed ration with a negative dietary cation–anion difference during the prepartum period. Pregnant dams underwent a drying-off process at approximately 215 ± 5 days of gestation. They were then transferred to the far-off pens and provided a far-off diet. Cows were then relocated to the close-up pens after about 255 days of gestation, where they were supplied with a close-up diet. Both far-off and close-up diets were delivered once daily. This procedure adhered to the farm’s established protocols. Animals were transferred to prepartum pens around 3 weeks before their anticipated calving date. A comprehensive description of the management practices for postpartum cows was documented before [19]. In brief, cows that showed signs of imminent calving were moved to a designated maternity pen. Within 1 to 2 h of calving, colostrum was collected, and the newborn calf was usually separated from its mother within 5–10 h after calving. The cows were kept in the maternity pen for a day after calving, during which they were monitored by the farm veterinarian for any health issues and treated in accordance with the standard practices of the farm. The cows confirmed healthy were transitioned to fresh-cow pens for approximately 3 weeks. Afterward, the cows were moved to lactation pens and fed a regular lactation diet. Diets provided during the transition period (i.e., far-off, close-up, and lactation) were formulated to meet the nutrient requirements specified in NRC [20] guidelines. Cows were milked thrice daily (0700, 1500, and 2300). About 6 weeks after parturition, the dams were enrolled in a double-Ovsynch protocol [19]. After 4 weeks, a timed artificial insemination was performed. Approximately a month later, pregnancy was examined using transrectal ultrasonography, and if negative, cows were subjected to timed artificial insemination using an Ovsynch protocol [19].

### 2.5. Management of Daughters

Newborn calves were managed similarly in both herds. In brief, calves were weighed using an electronic weighing scale (measurement precision = 0.1 kg) at birth and given 6 L of high-quality colostrum (a minimum Brix value of 22) using a nipple bottle in two feedings within 12 h after birth. These calves were individually housed in pens (1.5 m by 2.5 m), which were located within a roofed structure with open sides to allow natural light exposure. From 2 to 3 days of age, calves were fed pasteurized whole milk twice daily, which was shifted to once daily (morning feeding) from day 60 until 63 (weaning). Approximately 10 days after weaning, the calves were moved to a group-housed outdoor facility bedded with straw (15 animals/group) and were given a calf starter feed. The calves remained in these pens for about two months before being transferred to larger group pens (60 animals/group), where they were provided with a total mixed ration. Heifers initiated the synchronization and artificial insemination procedures when they reached approximately 55% of their mature body weight (±20–30 kg), typically above 13 months of age. The mature body weight in each herd was estimated annually by weighing a selected subset of mature cows, 40–50 in number (parity > 4 and days in milk > 100), post-milking, using a weighing scale with a measuring precision of 0.5 kg. The breeding program and housing conditions during and after pregnancy were managed similarly to dams (F_0_), as described above for dam management.

#### Binary-Coded Variables

The median value of AFC in the dataset was 706 days. The data were then converted into binary code, where AFC = 0 if it was equal to or less than 706, and AFC = 1 if it was greater than 706. The calving process was distinguished using a 4-level scoring system [21] as (1) easy calving, denoting situations where no assistance was needed; (2) calving needing minimal assistance; (3) calving process demanding demanded mechanical support; and (4) calving demanding caesarean section to facilitate the process. These data were binary-coded as 0 if the calving score was 1 or 2 and 1 if it was 3 or 4. Cows diagnosed with retained placenta were unable to discharge fetal membranes within 24 h after calving [22]. This was binary-coded as either absent (0) or present (1). Cows underwent uterine health evaluations by the farm veterinarian, and puerperal metritis was diagnosed within 21 days after calving [23]. The outcomes were then binary-coded as either absent (0) or present (1). Involuntary culling was defined from time from birth until culling. Culling decisions were mainly based on cow disorders (e.g., lameness, infectious diseases, digestive or metabolic disorders, or mastitis), reproductive inefficiency, or death. Animals sold mainly due to housing capacity or low production were excluded from the dataset to minimize the impact of voluntary culling on the analysis.

### 2.6. Statistical Analysis

Statistical analyses were run in SAS (version 9.4, SAS Institute Inc., Cary, NC, USA). To check the normality of residuals in the mixed linear regression model, the Shapiro–Wilk test was used. Additionally, we scrutinized the residuals for potential outliers and influential observations in each model. Outliers in calf birth weight and 305-day milk production dataset were defined as data points below or above 3 standard deviations from the mean [24]. These outliers, constituting an average of 2.51 and 4.06% of the initial data, respectively, were then discarded from the dataset. Initially, we built a full model that included the main factors of the herd, dam parity, and maternal summer season exposure during gestation, plus their two-way interactions, as follows:

Y_ijklm_ = μ + H_i_ + DP_j_ + MSS_k_ + (H × DP)_ij_ + (H × MSS)_ik_ + (DP × MSS)_jk_ + Y_l_ + A_m_ + e_ijklm_ (1)

where Y_ijkl_ = dependent variable; μ = the mean; H = the fixed effect of herd (A and B); DP = the fixed effect of dam parity (two-level variable: nulliparous or parous); MSS = the fixed effect of maternal summer season exposure during gestation (three-level variable: summer exposure during the first, second, or third trimester); (DP + MSS) = interaction between maternal summer season exposure and dam parity; (H × DP) = interaction between herd and dam parity; (H × MSS) = interaction between herd and maternal summer season exposure; Y = random effect of the year; A = random effect of animal; e = the residual error.

A manual backward stepwise elimination procedure was applied in order to identify significant explanatory variables; nonsignificant interactions (*p* > 0.10) between herd and dam parity or summer season exposure were excluded from the final model one by one. However, the interaction between maternal summer season exposure and dam parity remained in all models, irrespective of the significance level. The interaction of herd by dam parity or maternal summer season exposure was insignificant (*p* > 0.05) for any of the other outcomes evaluated in this study.

PROC MIXED in SAS was used to analyze continuous outcomes, including calf birth weight and 305-day milk production. In addition, calves were categorized based on a birth weight threshold of 35 kg, grouped as either <35 kg or >35 kg for further analysis (Table 1). Pairwise comparisons were conducted using the PDIFF option in SAS. Statistical significance threshold was set at *p* < 0.05. For the binary outcome variables (AFC, dystocia, retained placenta, and metritis), PROC GLIMMIX in SAS was used to estimate the odds ratio (associated with 95% confidence intervals), assuming a binary distribution with the logit link function. Multivariable Cox proportional hazard models were created in SAS (PROC PHREG) to evaluate the effects of dam parity and maternal summer season exposure during different gestational stages on the risk of culling from the herd (time to an event outcome). We used the Efron approximation (TIES = EFRON option) to handle tied event times in the data. The Wald chi-square test was employed to distinguish differences among the groups. The outputs were expressed as a hazard ratio (associated with 95% confidence intervals). A hazard ratio less than 1 implies a lower risk of removal from the herd, while a hazard ratio > 1 is indicative of a higher risk of removal from the herd (event occurring sooner).

## 3. Results

The effects of maternal summer season exposure during different gestational stages (early, mid, or late) and dam parity (2-level variable: nulliparous or parous dam) on calf birth weight are presented in Table 1. Regardless of dam parity, birth weight was lowest in female calves exposed to the summer season during the final trimester of their intrauterine development (*p* < 0.01). Female calves born to parous dams were generally heavier at birth than those born to nulliparous heifer dams. No significant interaction was found between maternal summer season exposure and dam parity on calf birth weight (*p* = 0.22).

Estimated odds ratios for the effects of dam parity and maternal summer season exposure during different gestational stages on AFC in the female offspring are presented in Table 2. The probability of AFC beyond the median day (706 days) tended to be greater (*p* = 0.08) in offspring born to parous vs. nulliparous dams. Maternal summer season exposure had no significant effect on AFC (*p* = 0.31).

Estimated odds ratios for the effects of dam parity and maternal summer season exposure during different gestational stages on the incidence of postpartum diseases in the offspring beginning as a first-lactation cow are presented in Table 3. Maternal exposure to the summer season at the different trimesters did not affect the incidence of dystocia, retained placenta, and metritis in the offspring (F_1_ generation). Daughters of parous vs. nulliparous dams were more likely to experience dystocia (odds ratio = 1.73; 95% CI = 1.49–1.99; *p* < 0.01) and metritis (odds ratio = 1.16; 95% CI = 1.03–1.31; *p* = 0.02) but less likely to experience retained placenta (odds ratio = 0.71; 95% CI = 0.57 to 0.89; *p* < 0.01).

Table 4 presents information on the effects of maternal summer season exposure during different gestational stages and dam parity on the first lactation 305-day milk production of the offspring as a first-lactation cow (F_1_ generation). Daughters born to nulliparous dams encountering the summer season in the first trimester produced, on average, 196 and 366 kg less milk throughout lactation than those in the second and third trimesters, respectively. There was no difference in the milk production between daughters born to parous dams exposed to summer in the first or second trimester (mean = 11,046 kg); both were lower than those exposed in the third trimester (11,321 kg; *p* < 0.01). No interaction existed between maternal summer season exposure and dam parity on the first lactation 305-day milk production (*p* = 0.91).

Information on the effects of dam parity and maternal summer season exposure during different gestational stages on the risk of culling (time from birth until removal from the herd) in the female offspring, using the multivariable Cox proportional hazard model, is presented in Table 5. The culling hazard was lower in the female offspring born to nulliparous heifer dams vs. those born to parous dams (hazard ratio = 1.17; 95% CI = 1.12–1.23; *p* < 0.01). The risk of culling in the female offspring decreased as their intrauterine exposure to the summer season moved from the first trimester to the third trimester (*p* = 0.01).

## 4. Discussion

Our assumption that the summer season (June to September) causes heat stress is supported by the THI values collected from the data over 11 consecutive years (Figure 2), revealing that the mean THI from June to September was 75.2. In addition, the farm management team reported an approximately 8–10% milk production decline during the summer season compared to the winter season in both herds. This direct effect of the summer season on lactation performance strengthens the assumptive deleterious impacts of summer heat exposure on pregnant dams.

### 4.1. Maternal Summer Season Exposure

In agreement with our findings that calves born to dams experiencing summer heat stress during the final trimester of pregnancy were lighter than those in the early stages, Makiabadi et al. [25] also reported that calves experiencing the summer season during the first trimester of intrauterine development were heavier at birth than those exposed in later developmental phases. The fetus experiences its most rapid growth during the third trimester, with approximately 60% of its final birth weight gained in the last two months of gestation [26]. During the later developmental stages, the fetus requires a substantial influx of nutrients and relies heavily on maternal nutrition to reach its full growth potential. Exposure to heat stress may diminish the supply of nutrients within the intrauterine environment, potentially compromising fetal development [12].

Our findings suggested that female offspring born to dams who experienced the first trimester of their pregnancy in the summer season had suboptimal production in their first lactation, regardless of their dam parity. The mammary gland begins to develop during the early stages of embryonic development [27], with the initial mammary anlage in bovine embryos becoming visible at about 32 days of gestation [28]. This milestone signifies the potential detrimental impact of early intrauterine hyperthermia on mammary gland function, potentially inducing molecular and cellular alterations with long-term consequences in the production capacity of the offspring [25,29]. As the pregnancy advances, the developing embryo becomes more resilient to temperature fluctuations, as enhanced resilience is created in response to the cellular damage caused by maternal heat stress [12,30]. This increased thermotolerance involves mechanisms such as enhanced protection from heat-shock proteins [31,32].

Monteiro et al. [7] reported that heifers who had experienced heat stress during their late prenatal development had lower milk production capacity in their first lactation (5.1 kg/d), likely because of alterations in the morphology of their mammary tissue compared to their counterparts whose dams were housed in cool conditions. In support of this, Pinedo and De Vries [3] reported the life-long consequences of conception season for the progeny and identified that a summer conception (July to September) vs. a winter conception (December to February) resulted in the offspring exhibiting lower 305-day milk yield capacity, likely because of the altered epigenome affecting the early embryo.

It is important to highlight that, in addition to impacting milk production capacity, the summer season might also affect the milk composition. However, we were unable to retrieve these data as the participating herds did not have a well-established system for recording milk composition during the data collection period. Future studies should further investigate this aspect.

### 4.2. Maternal Parity Effect

The lower birth weight of calves (F_1_) born to heifer dams as compared to parous dams could be explained by intrauterine growth restriction in the first-parity heifers, as a positive correlation exists between dam body weight and calf birth weight [33]. Duncan et al. [15] reported that dry total placental mass was approximately 20% lesser in primiparous vs. multiparous dams. During the final trimester of gestation, the fetus undergoes rapid growth. In the case of parous cows, they enter a nonlactating phase (the dry period) during the late stages of pregnancy. Meanwhile, pregnant heifers continue to grow throughout the entire duration of their gestation. This creates an altered nutrient partitioning hierarchy, which results in nutrient competition between the developing fetus and the growth requirements of the dam [34,35].

Increased incidence of dystocia and metritis in daughters (F_1_) of parous vs. nulliparous dams possibly indicates that dam age and dam health status can influence the in-utero developmental programming, with implications for postnatal phenotypic changes involved in health and production outcomes [35]. Contrary to data of dystocia and metritis, incidence of retained placenta was lower in daughters (F_1_) of parous vs. nulliparous. This observation indicates that alterations in the epigenome are not always linked to the development of negative postnatal phenotypes. Instead, certain epigenetic changes during prenatal development can manifest as adaptive benefits [36]. Carvalho et al. [35] identified that female offspring born to cows with clinical diseases during lactation exhibited a lower incidence of clinical diseases as young heifers and then during their first lactation. This observation was explained by the developmental origins of health and disease theory [37], suggesting that prenatal programming may represent an adaptive response to an adverse environment [38]. Placental detachment relies on collagen degradation and proteolysis, both orchestrated by maternal immune cells [39]. It is likely that heifers born to nulliparous vs. parous cows lacked the proper signals to activate the innate immune system, which is required for the timely expulsion of the placenta. However, we do not have data on the incidence of retained placenta in dams in their previous deliveries, and further data are necessary to better understand the underlying mechanisms involved.

In support of our finding that offspring born to nulliparous dams remained in the herd longer than those born to parous dams, Fuerst-Waltl et al. [40] also reported a negative association between maternal age and daughter longevity, speculating that chromosomal abnormalities and accumulated genetic damage over time may contribute to this observation. Increased involuntary culling forces the producers to be less able to keep the most productive animals in the herd, negatively affecting breed development and genetic improvement [41]. Cows with a shorter herd lifespan increase the cost of replacements, as heifer development to replace culled cows is a costly and expensive decision for cow–calf producers [42].

It is important to acknowledge that potential confounding effects can arise in retrospective longitudinal studies due to the nature of chronological analyses. Although we attempted, prior to designing this study, to choose the two herds to have a comparable number of animals as well as comparable feeding and health management protocols within a similar location, this study may suffer from the limitation that confounding factors beyond the summer season, such as diseases, parasitic infestations, and thus different management practices, may also have influenced the outcomes. This complexity highlights the need for conducting further investigations under controlled conditions to confirm the validity of these outcomes.

### 4.3. Future Perspective

This dataset allowed us to quantify the comparative effects of summer season exposure of dams during the early, mid, and late gestational stages as an environmental stressor and dam parity as a maternal factor on the future health and milk production capacity of the female offspring (F_1_ generation). In this observational study, we presented evidence supporting greater first-lactation milk loss and increased culling risk in female offspring exposed to summer during the first trimester of intrauterine development compared to later developmental stages. These preliminary findings may carry significant implications for adapting dairy herds to the rapidly changing climate, likely contributing to the long-term sustainability and profitability of the dairy industry. Addressing heat stress in utero during early pregnancy necessitates identifying viable management strategies to minimize environmental insults during this critical fetal development to enable further optimization of postnatal health and production outcomes of the offspring. Planning the timing of gestation to avoid summertime exposure of pregnant dams to the most sensitive period of their gestation appears to be an ideal management solution but may be challenging to implement in some production systems. Using heat-abatement strategies during early pregnancy could be a viable management strategy and is expected to produce healthier and more productive offspring in adulthood. Raising awareness of the detrimental effects of heat stress during early gestation on the future outcomes of the progeny and the associated economic losses would motivate producers to invest in heat-stress abatement measures.

## 5. Conclusions

In support of our hypothesis, the offspring experiencing the summer season during their early intrauterine development had less milk production capacity as a first-lactation cow than those that experienced the summer season during later developmental stages. The risk of culling also decreased as exposure to the summer season moved from the first to the third trimester. Dam parity was also identified as an important factor affecting the health of offspring, as daughters of parous vs. nulliparous dams were more likely to experience dystocia and metritis but less likely to experience retained placenta. The risk of culling was lower in the offspring born to nulliparous than those born to parous dams. Our results did not identify any interaction effect between summer season exposure at different gestational stages and dam parity on any of the outcomes assessed in the F_1_ generation. This study provides a valuable foundation for future studies and opens a new research area to investigate the biological mechanisms responsible for this association. Further research under controlled conditions is needed to identify molecular changes and physiological mechanisms during early embryonic and fetal development related to heat stress. It is important to determine the likely epigenetic effects occurring during the early stages of gestation in association with maternal heat stress that could affect the embryo at the early developmental stage and the potential implications in the future outcomes of the offspring. Long-term investigations under controlled conditions are suggested to identify the potential impact of maternal heat stress during early intrauterine development on reproductive performance of F_1_ generation. Future investigation should focus on assessing the potential impacts of the summer season and dam parity on the performance and health of granddaughters (i.e., the F_2_ generation or the offspring of the F_1_ generation).

## Figures and Tables

**Table 1:** Effects of maternal summer season exposure during different gestational stages and dam parity on birth weight (kg) of female calf (F_1_ generation).

Category	*N*	Gestation Exposure in Summer			*p*-Value
		First Trimester	Second Trimester	Third Trimester	
*Dam parity*					
Nulliparous	4525	37.2 ± 0.14 ^a^	37.5 ± 0.12 ^a^	36.6 ± 0.10 ^b^	<0.01
Parous	6321	38.6 ± 0.13 ^a^	38.7 ± 0.10 ^a^	38.2 ± 0.09 ^b^	<0.01
*Birth weight categorization*					
<35 kg	3084	32.4 ± 0.12	32.1 ± 0.10	32.1 ± 0.08	0.06
>35 kg	7762	40.0 ± 0.07 ^a^	40.1 ± 0.06 ^a^	39.8 ± 0.05 ^b^	0.01

Values are estimated least squares mean (±SE). ^a,b^ Different letters within each row indicate statistical difference.

**Table 2:** Estimated odds ratios for the effects of dam parity and maternal summer season exposure during different gestational stages on risk of calving with a longer AFC * in the female offspring (F_1_ generation).

Category	*N*	Incidence (%)	Odds Ratio (95% CI)	*p*-Value
*Dam parity*				
Nulliparous	3740	48.5	Referent	0.08
Parous	4943	49.6	1.09 (0.99–1.19)	
*Gestation exposure in summer*				
First trimester	1829	50.4	Referent	0.31
Second trimester	2834	49.1	0.92 (0.81–1.03)	
Third trimester	4020	48.6	0.93 (0.83–1.04)	

* Median age at first calving (AFC) was 706 days. The dataset was converted into binary code, with AFC = 0 if equal to or less than 706 and AFC = 1 if greater than 706. CI = confidence interval.

**Table 3:** Estimated odds ratios for the effects of dam parity and maternal summer season exposure during different gestational stages on the incidence of postpartum diseases in the female offspring beginning as a first-lactation cow (F_1_ generation).

Category	*N*	Incidence (%)	Odds Ratio (95% CI)	*p*-Value
*Dystocia*				
*Dam parity*				
Nulliparous	3740	8.50	Referent	<0.01
Parous	4943	14.0	1.73 (1.49–1.99)	
*Gestation exposure in summer*				
First trimester	1829	11.7	Referent	0.30
Second trimester	2834	12.5	1.01 (0.84–1.23)	
Third trimester	4020	11.1	0.90 (0.75–1.08)	
*Retained placenta*				
*Dam parity*				
Nulliparous	3734	4.93	Referent	<0.01
Parous	4927	3.67	0.71 (0.57–0.89)	
*Gestation exposure in summer*				
First trimester	1819	4.95	Referent	0.39
Second trimester	2827	4.03	0.85 (0.64–1.13)	
Third trimester	4015	4.01	0.84 (0.64–1.09)	
*Metritis*				
*Dam parity*				
Nulliparous	3727	17.1	Referent	0.02
Parous	4916	18.1	1.16 (1.03–1.31)	
*Gestation exposure in summer*				
First trimester	1813	17.7	Referent	0.67
Second trimester	2823	17.4	0.94 (0.80–1.10)	
Third trimester	4007	17.8	0.99 (0.86–1.15)	

CI = confidence interval. An odds ratio less than 1 implies a decreased likelihood of the outcome compared to the referent group, and an odds ratio > 1 indicates an increased likelihood of the outcome.

**Table 4:** Effects of maternal summer season exposure during different gestational stages on the 305-day milk production (kg) of the offspring as a first-lactation cow (F_1_ generation) according to their dam parity.

Dam Parity	*N*	Gestation Exposure in Summer			*p*-Value
		First Trimester	Second Trimester	Third Trimester	
Nulliparous	2261	10,998 ± 160 ^c^	11,194 ± 158 ^b^	11,364 ± 154 ^a^	<0.01
Parous	2805	10,973 ± 159 ^b^	11,119 ± 155 ^b^	11,321 ± 152 ^a^	<0.01

Values are estimated least squares mean (±SE). ^a–c^ Different letters within each row indicate statistical difference.

**Table 5:** Multivariable Cox proportional hazard model evaluating the effects of dam parity and maternal summer season exposure during different gestational stages on the risk of culling in the female offspring (F_1_ generation).

Category	*N*	Hazard Ratio	95% Confidence Interval	*p*-Value ^1^
*Dam parity*				
Nulliparous	4525	Referent		<0.01
Parous	6321	1.17	1.12–1.23	
*Gestation exposure in summer*				
First trimester	2345	Referent		0.01
Second trimester	3513	0.96	0.90–1.02	
Third trimester	4988	0.90	0.84–0.95	

^1^ The *p*-Values from Type III Wald chi-square test.

## Data Availability

None of the data used in this study were deposited in an official repository. Data are available from the corresponding authors upon request.

## Supplementary Materials

The following supporting information can be downloaded at https://www.mdpi.com/article/10.3390/ani14203040/s1, Table S1: Characteristics and performance metrics of the participating herds. Figure S1: Box-and-whisker plot of monthly average temperature and relative humidity from 2013 to 2023.
