# Supplementary material for: Does Exposure to Summer Season at Different Stages of Intrauterine Development and Maternal Parity Affect Health and First-Lactation Milk Production of Female Offspring of Holstein Cows?
Source: Animals (Basel). 2024 Oct 21;14(20):3040. doi: 10.3390/ani14203040 (PMC11504960; doi:10.3390/ani14203040)
Supplement: Supplementary file 1 [file animals-14-03040-s001.zip › animals-3253710-supplementary.pdf]

Supplementary Table S1. Characteristics and performance metrics of the participating herds.

Items	Herd A	Herd B
Herd size	5,400	5,800
Proportion of lactating cows, %	42.6	43.1
No. of animals enrolled in the study	5,329	5,517
Proportion of nulliparous dams, %	42.7	41.5
Proportion of parous dams, %	57.3	58.5
Calf birth body weight, kg	38.0	37.7
Age at first calving, d	713	724
Dry matter intake, kg/day*		
Close-up period	15.3	14.9
Postpartum period (calving to 21 DIM)	21.5	20.3
Incidence of postpartum diseases[1], %		
Dystocia	12.2	11.1
Retained placenta	3.24	5.14
Metritis	17.2	18.1

* Data on dry matter intake mainly from 2020 through 2023.

DIM = days in milk.

[1] Disease incidence of F_1 generation.

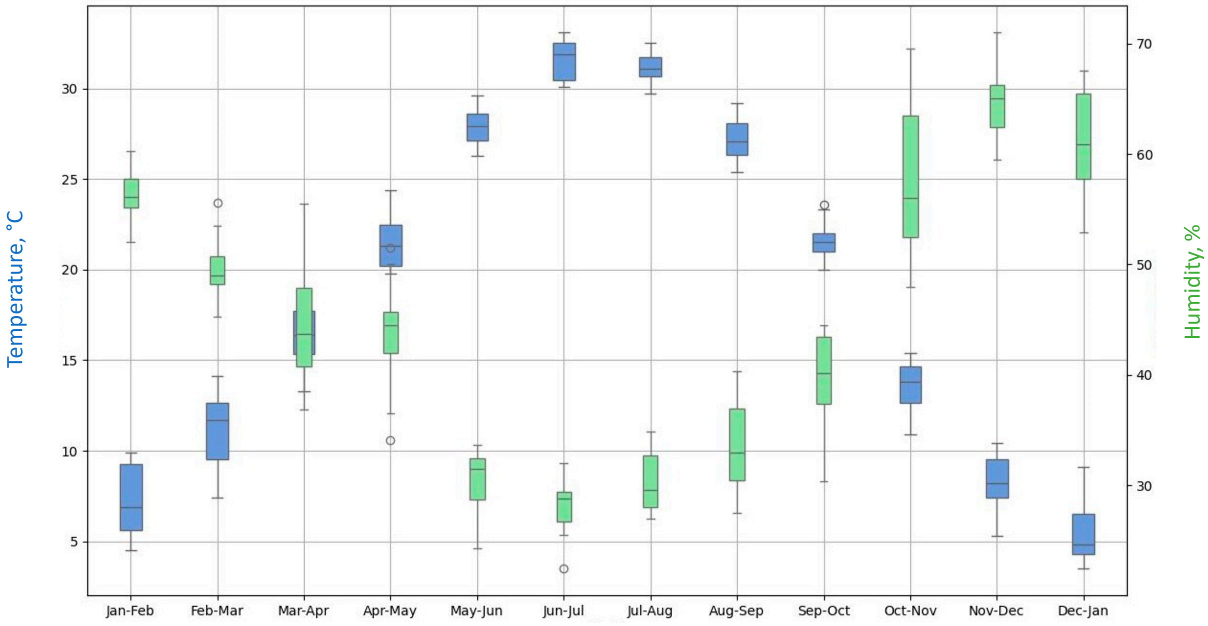

Supplementary Figure S1. Box-and-whisker plot of monthly average temperature and relative humidity from 2013 to 2023.
